# Intelligent Diagnostic Prediction and Classification System for Chronic Kidney Disease

**DOI:** 10.1038/s41598-019-46074-2

**Published:** 2019-07-03

**Authors:** Mohamed Elhoseny, K. Shankar, J. Uthayakumar

**Affiliations:** 1 Faculty of Computers and Information, Mansoura University, Mansoura, Egypt; 2 School of Computing, Kalasalingam Academy of Research and Education, Krishnankoil, India; 3 Department of Computer Science, Pondicherry University, Pondicherry, India

**Keywords:** Chronic kidney disease, Diagnosis

## Abstract

At present times, healthcare systems are updated with advanced capabilities like machine learning (ML), data mining and artificial intelligence to offer human with more intelligent and expert healthcare services. This paper introduces an intelligent prediction and classification system for healthcare, namely Density based Feature Selection (DFS) with Ant Colony based Optimization (D-ACO) algorithm for chronic kidney disease (CKD). The proposed intelligent system eliminates irrelevant or redundant features by DFS in prior to the ACO based classifier construction. The proposed D-ACO framework three phases namely preprocessing, Feature Selection (FS) and classification. Furthermore, the D-ACO algorithm is tested using benchmark CKD dataset and the performance are investigated based on different evaluation factors. Comparing the D-ACO algorithm with existing methods, the presented intelligent system outperformed the other methodologies with a significant improvisation in classification accuracy using fewer features.

## Introduction

In the last decade, the recent developments in information technology include mobile communication system, big data, Internet of Things (IoT), and wearable computing are employed in the field of healthcare. In particular, various intelligent healthcare systems are modeled with the help of big data and mobile computing devices to offer intellectual and expert services. Also, the increase in medical data leads to different issues for managing, storing and processing data. Persistent, low-grade inflammation is now treated as an important characteristic of chronic kidney disease (CKD). Though considerable enhancements have been made in the healthcare domain, CKD still being a critical health problem which affects 10–15% of the population, and its pervasiveness is continuously increasing. Because of its subtle nature, CKD is not often identified in premature stages^1^. A person with CKD has a higher chance of developing heart disease^2,3^. The earlier stage of CKD does not show any major symptoms and it very hard to identify it without some tests like urine and blood test. When the CKD is detected at the initial stages, preventive actions and better treatment can be given to control the chances of dialysis or transplantation. A study reported that the earlier detection of CKD could reduce the growth of disease even by the nurses in the specialization of nephrology and primary care doctors^4^. Generally, imaging techniques are employed to identify the presence of CKD. But, because of a large number of patients, it is impossible to test each person, and people with a higher possibility of having CKD will be recommended to undergo extensive testing. At present, the preservation of clinical database becomes a difficult process in the healthcare industry. The patient’s data holds different features and diagnosis related to disease needs to be provided with extreme importance to attain high quality service. Since the data archived in the hospital database may have missing as well as unnecessary data, it becomes burdensome to mine the patient data. So, better data processing and data reduction approaches are needed prior to the application of data mining techniques. Then, the identification of CKD becomes simpler and faster when the available data is accurate and reliable.

Identification of CKD from the patient’s data can be treated as a data classification issue. The classification task is generally a supervised learning process that deduces a connection between features and class labels. A classification and prediction technique^5,6^ utilizes the training data to create a model and is applied for test data to analyze the prediction performance. Recently, artificial intelligence (AI) methodologies can be used to improve the available classification model. At the same time, the existence of several features in the high-dimensional medical data resulted in different problems such as overfitting, high computation complexity and low interoperability of the finishing model^7,8^. The easiest method to resolve the problem is to decrease the number of features using Feature selection (FS) approach. This procedure intends to select a feature subset by the elimination of redundant or irrelevant features. It is based on the idea of extracting the maximum possible information using a reduced number of features to save computation time^9^. The selected feature subset finds helpful in representing a classification function that has a serious impact on learning time, classification accuracy and cost involved with the features^10^. FS methods are applied in diverse applications such as data mining, ML and pattern recognition, to lessen the number of features for improving the prediction results^11^. Concerning the validation parameters, FS methods are classified into the wrapper, embedded and filter based approaches^12^. Filter approaches validate the feature subset by the use of fixed measures instead of learners and selected features^13,14^.

In contrast, the wrapper method makes use of the learning technique as a sub-process of evaluation for assessing the betterment of the chosen feature set. Even though wrapper methods are commonly employed, it facts few difficulties such as high computation complexity, identifying user-defined parameter of the learner, and inbuilt learner constraints^15^. Embedded approaches have less complexity compared to wrapper approaches. However, the chosen feature subset is based on the learning methodology^16^. Naturally, the embedded method integrates the filter and wrapper method and eliminates the limitations of them. Though the filter methods have low computation complexity, the chosen feature subset showed inadequate reliability for classification. Contrastingly, the wrapper methods attain higher classification performance with high computational complexity. These three methods have enhanced the features’ discrimination for classification. Furthermore, the process of FS has not improved the classifier but improved the features. In addition, the wrapper method and hybrid methods attained high classification performance but with the cost of high computational complexity.

To overcome these issues, we propose a novel wrapper approach for CKD identification by incorporating density-based FS (DFS) with Ant Colony based Optimization (ACO) named as D-ACO algorithm. The DFS approach is a heuristic method to evaluate the worthiness of a feature. The inclusion of DFS removes the unnecessary features and assists to increase the accuracy of the ACO based classifier and thereby improvise the final classifier results of D-ACO algorithm. The D-ACO algorithm is employed to a benchmark CKD dataset from UCI repository. For comparison purposes, ACO, genetic algorithm (GA) and particle swarm optimization (PSO) based classification algorithms are employed. The experimental outcome depicted that the presented D-ACO approach achieves effective classification performance over the compared approaches.

The upcoming sections of the study are planned as follows: Section 2 briefs the approaches relevant to the presented model. Section 3 discusses the presented D-ACO model, and section 4 investigates the results obtained by the D-ACO algorithm against CKD dataset. And, the concluding remarks are made in section 5.

## Related Works

Different techniques have been proposed for effective prediction of CKD by the exploitation of patient’s medical data. A Cuckoo Search trained neural network (NN-CS) method is presented for the identification of CKD^17^. Initially, the presented model is designed to resolve the issues that exist in the local search based learning methods. The CS algorithm helps to optimally select the input weight vector of the NN to train data properly. The classifier results of the proposed algorithm showed that it attains better performance. A modified version of NN-CS (NN-MCS) algorithm^18^ is developed to overcome the problem of local optima of the NN-CS algorithm. As the initial weights of the neuron connection control the NNs performance, the proposed method uses employs MCS algorithm to decrease the root mean square error (RMSE) value employed in the training process of NN. The simulation results reported that NN-MCS algorithm attained better performance than NN-CS method.

In^19^, two fuzzy classifiers are known as fuzzy rule-building expert system (FuRES) and fuzzy optimal Associative Memory (FOAM) are presented for the identification of CKD. FuRES generates a classification tree which comprises a minimal NN. It creates the classification rules to determine the weight vector with the least fuzzy entropy. The two fuzzy classifiers are employed for the identification of 386 CKD patients. Also, FuRES is better compared to FOAM especially in situations where the training, as well as the prediction process, contain a similar intensity of noise. FuRES and FOAM attained better performance in the identification of CKD; at the same time, FuRES is proficient than FOAM. In^20^, another fuzzy-based method is presented to identify the CKD. The author designed an Improved Hybrid Fuzzy C-Means (IHFCM), an improved version of FCM with Euclidean distance for the detection of CKD. This study revealed that the probability based methods are unsuitable for CKD prediction because of the necessity of proper output. Statistical methods, Bayesian classification or association rule based prediction methods are infeasible to use as it leads to inaccurate results. So, IHFCM is developed for the identification of CKD. At the initial stage, IHFCM removes the frequent records as a preprocessing step. Then, it computes the diffuse score for each value in the particular table of contents of the query. The higher fuzzy score represents the clusters of higher risk and lower fuzzy score indicates lower or no risk at all.

In the year 2017, Dilli Arasu *et al*.^21^ devised a novel method namely Weighted Average Ensemble Learning Imputation (WAELI). The missing values in the dataset reduce the precision level of CKD. As the existing methods use of data preprocessing technique, the data cleaning process is needed to fill up the missing values and to remove the inaccurate values. A recalculation procedure is present in different CKD stages where the missing values are computed and placed in their respective positions. Although the existing methods are effective, it needs an expert in healthcare dataset to ensure the values for CKD.

FS process acts as a significant part in the area of data classification, employed to find out a smaller set of rules from the training dataset with fixed goals. Different methodologies like AI techniques, bio-inspired algorithms are used for FS. In^22^, a wrapper method is presented by the hybridization of GA with support vector machine (SVM) called GA-SVM method to properly select the feature subset. The reduction in the redundant features of the proposed method improves the classification performance which is validated using five different disease dataset.

Naganna Chetty *et al*.^23^ also presented a wrapper method for CKD identification by following three steps: (1) a framework is generated from data mining, (2) Wrapper subset attribute evaluator and best first search approach are employed to select attributes and (3) Classification algorithms are employed. The experimental observations revealed that the accuracy is improved for reduced dataset compared to the original dataset^24^. developed a framework for enhancing the quality of CKD. This framework involves three processes include FS, ensemble learning and classification. The integration of Correlation-based FS (CFS) and k-nearest neighbor (kNN) classifier results in high classification accuracy^25^. developed another CKD identification method by the use of filter as well as wrapper approaches. The simulation outcome depicted that the decrease in a number of features does not ensure effective classification performance.

## Proposed Approach

**Figure 1:**
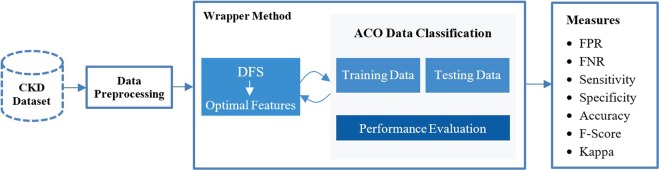
Block diagram of D-ACO algorithm.

**Figure 2:**
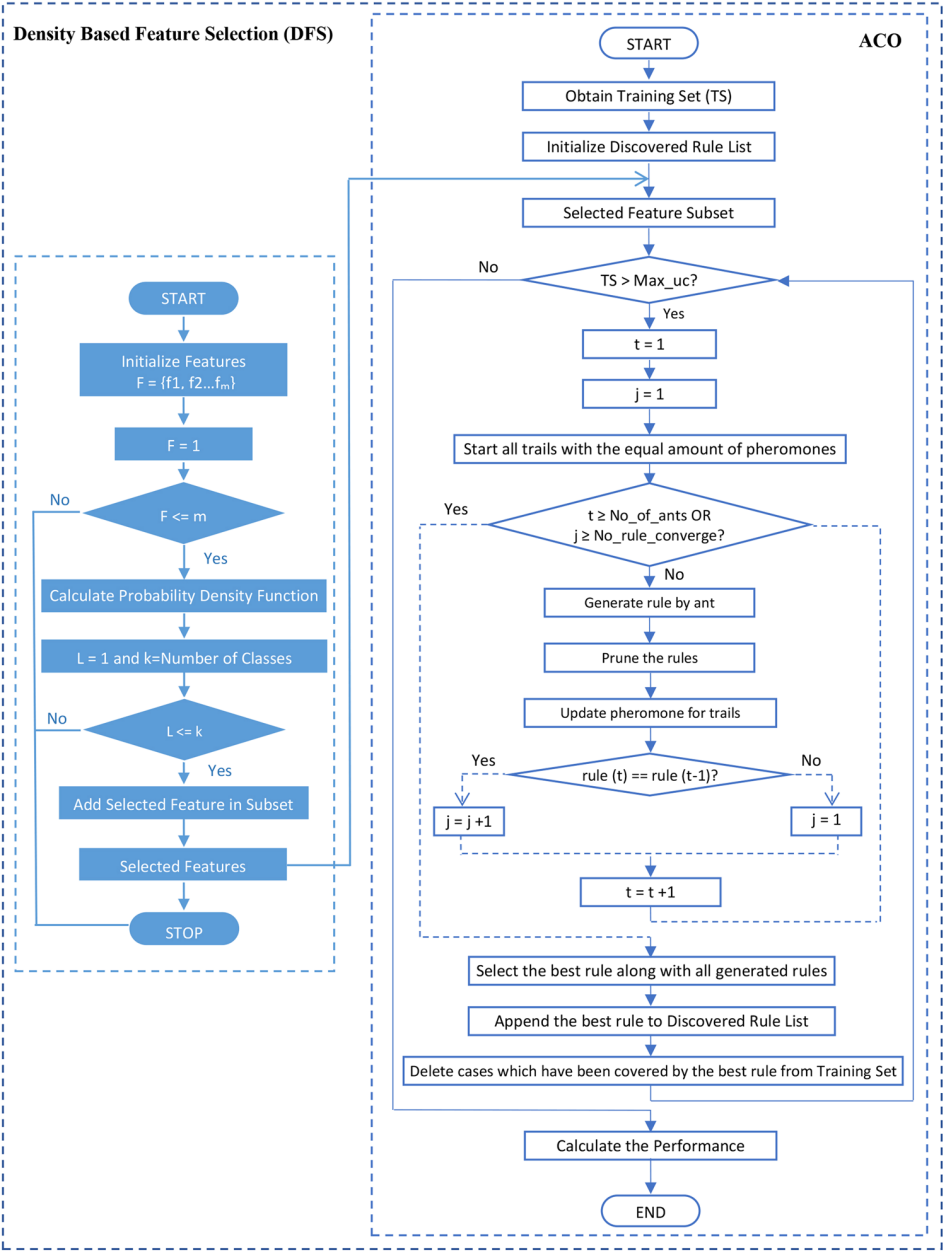
Flowchart of the D-ACO method.

The outline of the D-ACO algorithm is illustrated in Fig. 1. The proposed work operates on three stages: preprocessing, FS and classification. The preprocessing stage is the primary process since the database may contain redundant and noise data. By examining the data, different processes take place such as data cleaning, filling missing values, removing excessive data because the missing values and excessive data degrade the performance. In this work, a total of 24 features exists, and a few features are selected with the help of DFS. The purpose of using a wrapper method is the selection of best feature subset by repeatedly generating a set of features till the best subset is obtained by DFS. To register the obtained feature vector, ACO based classification approach is employed for classifying the data as the presence of CKD or absence of CKD. This combination of DFS and ACO in D-ACO algorithm allows the user to foresee and diagnose the health using their medical data. The proposed D-ACO model will attain high classification performance with few features and achieve optimal performance measurements. Moreover, the process involved in the D-ACO algorithm is shown in Fig. 2, and the pseudo code is provided in Algorithm 1 with the parameter settings in Table 1.

**Table 1:** Parameter Settings.

D_T_ ← Training Set
rule_list ← NULL list
Max_UC ← Maximum amount of uncovered cases
t ← Ant index
j ← Convergence of ants
τ ← Initialize equal pheromone trails
rule_best_ ← Add the optimal rule to Discovered Rule List
rule_t_ ← Ant_t_ construct the rules in an iterative manner
ConstructRule () ← Ant_t_ start with a null rule and incrementally generates the classification rule_t_ by totalling single term at a time to the existing rules
Prune_Rule (rule_t_) ← Removing unnecessary rules
Update_Pheromone () ← Pheromone updation of all trails

### Preprocessing

To provide effective performance with low cost for data mining processes, the quality of the data should be good. The values missing in the database should be filled in the whole CKD dataset. In some situations, when continuous features exist, the methods can be synchronized to build discrete traits. They contain some noisy and missing values in each instance. For improving the behavior of medical data, the original data is preprocessed^26^.

### Optimal FS

For the selection of optimal features, the proposed algorithm uses the following processes. Once the preprocessing of CKD dataset is completed, the next step is to organize the input data into groups. Here, DFS is used which selects a group of features in every iteration. A subset of the optimal features from the raw dataset is considered as the most significant feature for the classification process.

The DFS method is a heuristic approach used to evaluate the merits of features. The main assumption of considering a feature is good when every class has less overlap with the remaining classes. For the exploration and assignment of ranks, the DFS algorithm considers the distribution of features overall classes along with their correlation. The initial step of DFS is to compute the probability density function (PDF) of every feature in every class individually. And, the next step is the ranking procedure of the features based on the overlapping area. The common approaches to compute PDF can be divided into parametric and non-parametric approaches^27^. The former method assumes that the data follows Gaussian distribution and therefore the density estimation task is just to decide proper values for mean as well as the variance of the distribution. Contrastingly, non-parametric approaches have no assumptions regarding the shape of the density function; instead, it computes the density straightly from the instances. It is noted that many of the pattern recognition applications have no fixed format to estimate the density of the primary data. On the other hand, non-parametric methods can be utilized with random distributions with no consideration that the form of the fundamental densities are known^28^. Hence, the proposed method uses the parametric approach and is equated as

1 $$p(x)\cong \frac{k}{NV}$$

where, $p(x)$ represents the value of obtained PDF for instance $x$, $V$ is the volume around $x$, $N$ is the total number of instances and k is the number of instances in $V$. The determination of precise PDF can be found with increased $N$ and decreased $V$. The succeeding stage after the estimation of PDF in every class is to explore the worthiness of the feature using the calculated PDFs among the classes. As explained earlier, a feature is said to good when it every class has less overlap with the remaining classes. For the estimation of the quantity of the overlap among instances of classes of a particular features, PDF estimations for every feature and class label is used. After the increment in the overlapping area for a feature, the significance for the prediction of class label is decreased and its consideration leads to degraded classification performance. The overlapping value for a feature *f*, in class *cl* is computed using Eq. (2).

2 $${\rm{Overlapping}}({\rm{f}},{\rm{cl}})={\int }^{}\,{\rm{Min}}({\rm{PDF}}({\rm{cl}}),\,{\rm{Max}}({\rm{PDF}}({{\rm{cl}}}_{{\rm{j}}})))\,$$

where 1 ≤ j ≤ num_classes_ and j ≠ cl.

### Classification of CKD

For classification task, the ACO algorithm is employed for the extraction of classification rules, using the behavior of ant colonies and data mining techniques. This approach intends to allocate each instance to a class from the collection of predefined class, using the values of some features^29^. In general, the discovered knowledge in the classification process is defined in Eq. (3).

3 $${\rm{IF}} < conditions > THEN < class > $$

The rule predecessor (IF part) holds a set of conditions which is connected by a logical conjunction operator (AND). Then, the rule subsequent (THEN part) indicates the predicted classes for cases whose predictor features fulfill each term represented in the rule antecedent.

**Algorithm I:**
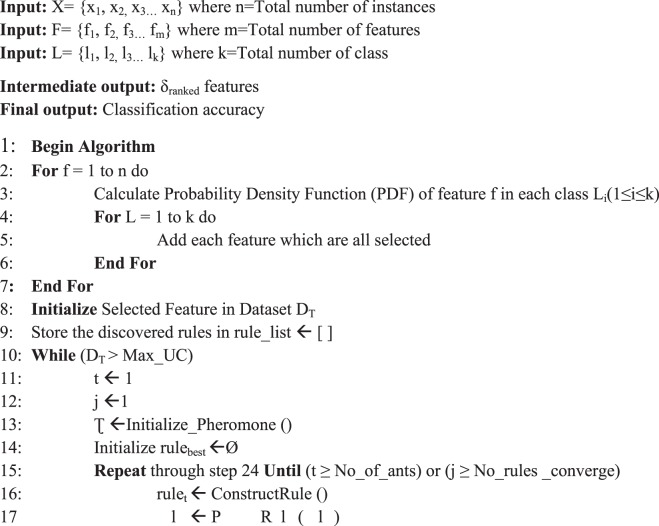
Density based feature selection with Ant Colony Optimization (D-ACO) for Data Classification

The application of ACO algorithm in the classification task of CKD involves the following processes:

- Structural schema
- Generation of rules
- Heuristic function
- Pruning of rules
- Pheromone update
- Utilizing discovered rules

#### Structural schema

**Figure 3:**
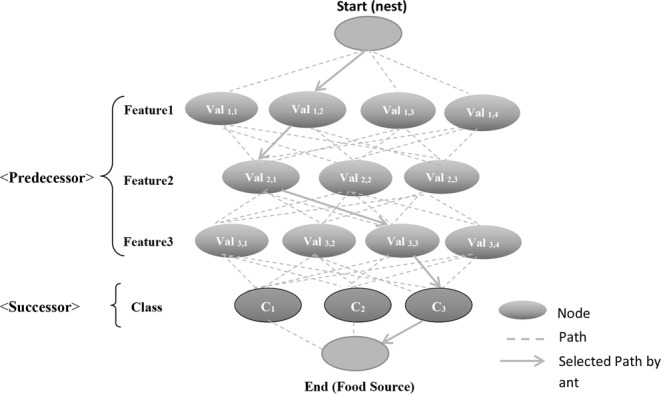
Structural schema of ACO.

The structural schema of the presented classification model is represented in Fig. 3. The artificial ants begin the traversal from the start node on the top that is considered as a virtual nest. The lower level nodes represent different features and every feature holds a number of values. A feature can be defined as *f*_*m*_ and V_mn_ is the discrete value belongs to the feature, where $\,i$ denote the series number of features and *j* denotes the series number of the value present in the feature. The end of the features is the class and the class values are written as C_k_, where k is the sequence value in the class. As shown in figure, the ant starts its traversal from the source and picks a value for the class and consumes the artificial food. When the traversal process is completed, a value will be chosen for every class. To discover the rules, sufficient number of ants trail an identical path as discussed below. In this case, as shown in Fig., the discovered path is indicated by solid line: Start-Val_1,2_-Val_2,1_-Val_3,3_-C_3_-End.

#### Rule generation

As explained earlier, the ants begin from the artificial nest and selects a value for every feature for rule generation. This task is performed using the probability function as provided in Eq. (3). It provides the probability (P_mn_) that V_ij_ is chosen as value of f_m_ (f_m_ = V_mn_) where f_m_ is the m^th^ feature and V_ij_ is the n^th^ value of the feature.

3 $${{\rm{P}}}_{{\rm{mn}}}=\frac{{{\rm{\eta }}}_{{\rm{mn}}}.{{\rm{\tau }}}_{{\rm{mn}}}}{{\sum }_{m=1}^{a}({{\rm{x}}}_{{\rm{i}}}).{\sum }_{n=1}^{b}({{\rm{\eta }}}_{{\rm{mn}}}.{{\rm{\tau }}}_{{\rm{mn}}}({\rm{t}}))}\,$$

where ${{\rm{\eta }}}_{{\rm{mn}}}\,\,$is the problem dependent heuristic function for V_ij_ and ${{\rm{\tau }}}_{{\rm{mn}}}\,\,$indicates the quantity of pheromone.

For discovering the classifier rules, a sequential covering approach is used. Initially, the discovered rule count is kept as NULL and the training set holds the identified rules. On the discovery of rules at each iteration, the identified rules will be moved to the classification rule list and removed from the training set. The rule discovery process will be carried out when any one of the following conditions is satisfied.

i. The number of cases should be lesser than the fixed value can be placed to the rule, named as minimum_cases_per_rule.
ii. After the exploitation of all features, the rule generation task gets stopped. The ants employ a probability function (P_mn_) for selecting a feature value for rule generation as represented in Eq. (3).

#### Heuristic function

For each term_mn_, ACO algorithm calculates the heuristic function ${{\rm{\eta }}}_{{\rm{mn}}}\,\,$of a that defines the quality of this term based on the capability to enhance the predictive results of the rule^30^. Particularly, the value of ${{\rm{\eta }}}_{{\rm{mn}}}\,\,$for term_mn_ indicates a measure of the entropy integrated with that term. The entropy will be determined for each term_mn_ as given in Eq. (4).

4 $$H(W|{A}_{m}={V}_{mn})=-\,\sum _{w=1}^{k}(P(w|{A}_{m}={V}_{mn}).lo{g}_{2}P(w|{A}_{m}={V}_{mn}))$$

where W indicates the class feature, k is the number of classes and $P(w|{A}_{m}={V}_{mn})$ is the empirical probability of observing class *w* conditional on having observed *A*_*m*_ = *V*_*mn*_.

#### Rule pruning

It is a commonly employed approach for eliminating unwanted terms which exist in the rule. It considerably improvises the classifying ability of the rule and assists to resolve the issue of overfitting of the training data. Once the rule construction process gets completed, the rule pruning procedure will begin. It eliminates the pointless rules produced by ants in every step, which improves the rule quality. The value of rule quality (Q) is present in the range of 0 ≤ *W* ≤ 1as provided in Eq. (5).

5 $$Q=\,\frac{TP}{(TP+FN)}\ast \frac{TN}{(FP+TN)}$$

where TP- True positive, TN- True Negative, FN- False Negative and FP- false positive.

#### Pheromone update

The pheromone updating procedure indicates the volatility of ant pheromone in the physical world^31,32^. Due to the positive feedbacking procedure, the errors in the heuristic measure could be corrected and results to enhanced classifier performance. The ants apply this process to determine better classifier rules. At the beginning, every trail is provided with equal quantity of pheromone as represented in Eq. (6).

6 $${{\rm{\tau }}}_{{\rm{mn}}}(t=0)=\frac{1}{{\sum }_{m=1}^{a}{b}_{m}}$$

where a_i_ indicates feature count and b_m_ is the likely values of a_m_. The quantity of pheromone on the nodes has been utilized by the current rule gets updated due to the pheromone deposition by the ants in the process of path discovery. At that instant, the pheromone evaporation is also needed to be defined. As a result, the iterative operation is also carried out using Eq. (7).

7 $${{\rm{\tau }}}_{{\rm{mn}}}({\rm{t}})=(1-{\rm{\rho }}){{\rm{\tau }}}_{{\rm{mn}}}({\rm{t}}-1)+(1-\frac{1}{1+{\rm{Q}}}){{\rm{\tau }}}_{{\rm{mn}}}({\rm{t}}-1)$$

where $\,{\rm{\rho }}$ is the pheromone evaporation rate, Q is the quality as represented in Eq. (7) and t is the sequence number of the iteration. In contrast, the nodes which has not utilized by the current rule will have only pheromone evaporation as given in Eq. (8).

8 $${{\rm{\tau }}}_{{\rm{mn}}}({\rm{t}})=\frac{{{\rm{\tau }}}_{{\rm{mn}}}({\rm{t}}-1)}{{\sum }_{{\rm{m}}=1}^{{\rm{a}}}{\sum }_{{\rm{n}}=1}^{{{\rm{b}}}_{{\rm{i}}}}{{\rm{\tau }}}_{{\rm{mn}}}({\rm{t}}-1)}$$

Eq. (8) defines that the quantity of pheromone of undiscovered nodes gets decreased with an increase in time.

#### Usage of discovered rules

To classify new instances, the identified rules are applied in the order as they were explored, as they are saved in an ordered list. The primary rule which conceals the new instance represents that the case is allocated by the class identified by the rule’s resultant. When the new instance does not come under any of the rules in the list, the new instance undergoes classification using a default rule that identifies the important class in the collection of uncovered training cases.

## Performance Analysis

For the validation of the proposed D-ACO algorithm, it is simulated in MATLAB R2014a using Windows 10 operating system operating on a general-purpose PC with 8GB of RAM and an Intel i7 core running at 2.70 GHz.

### Dataset

**Figure 4:**
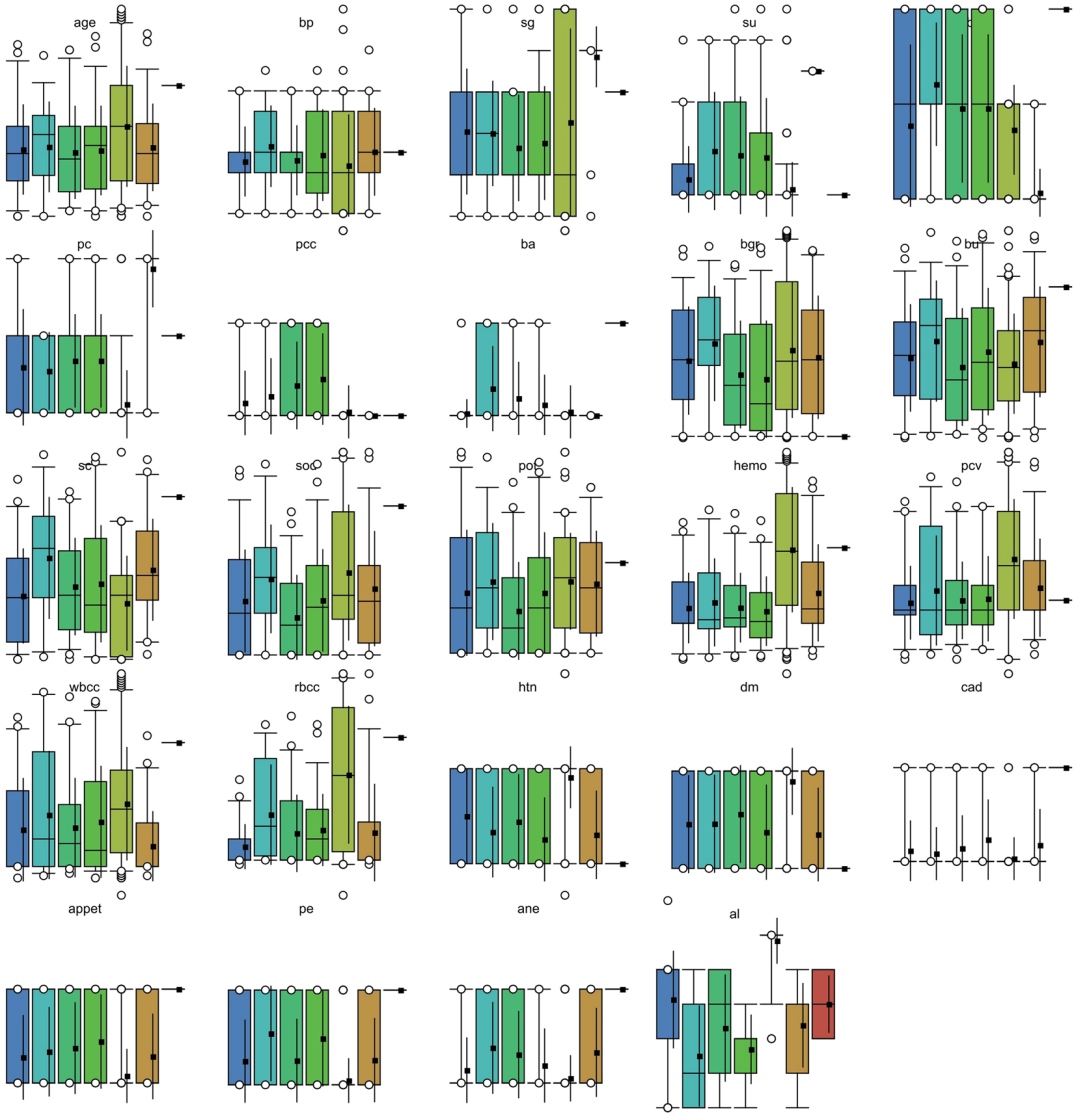
Sample frequency distribution of 24 features.

**Figure 5:**
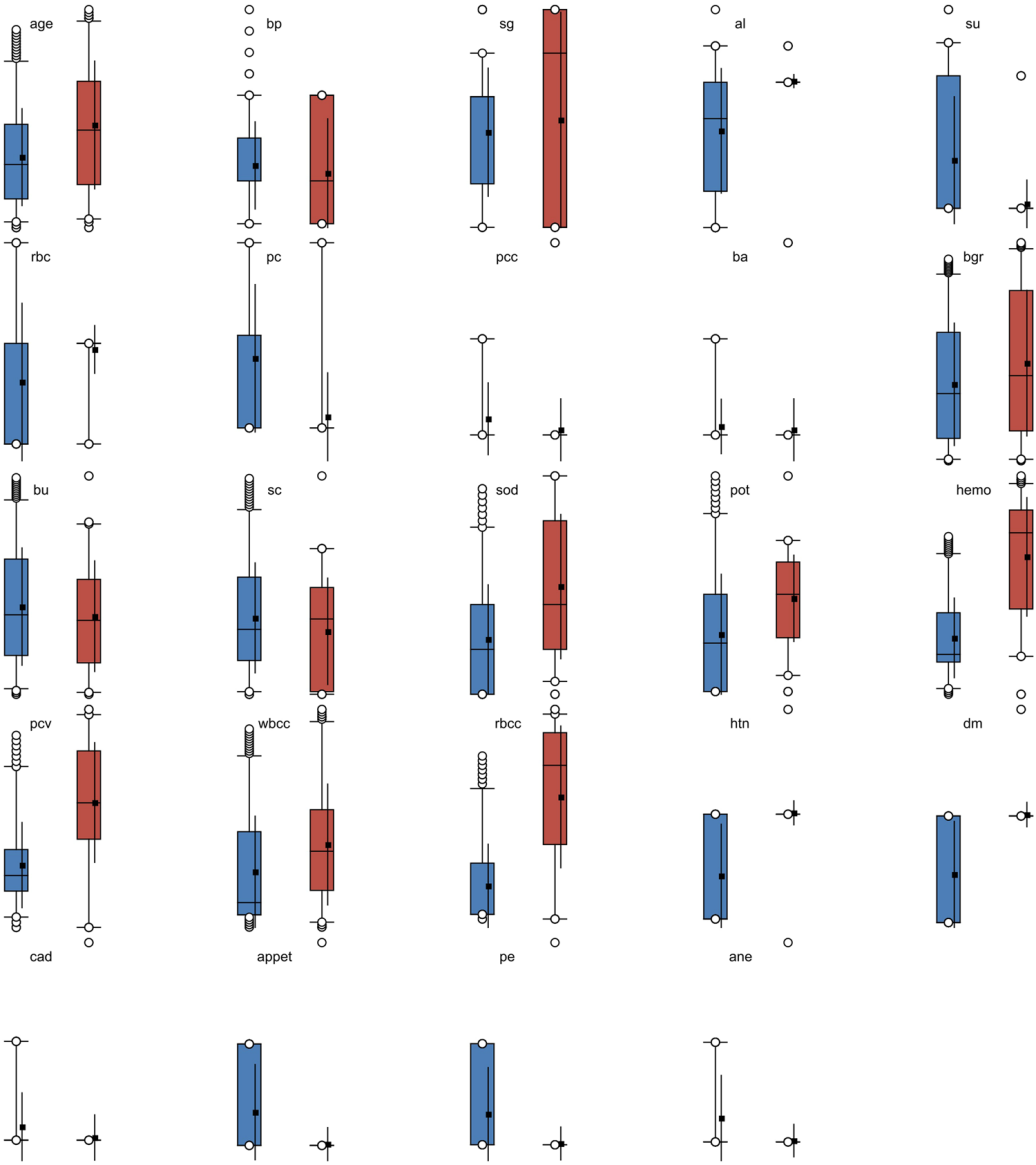
Sample class distribution of 24 features.

**Figure 6:**
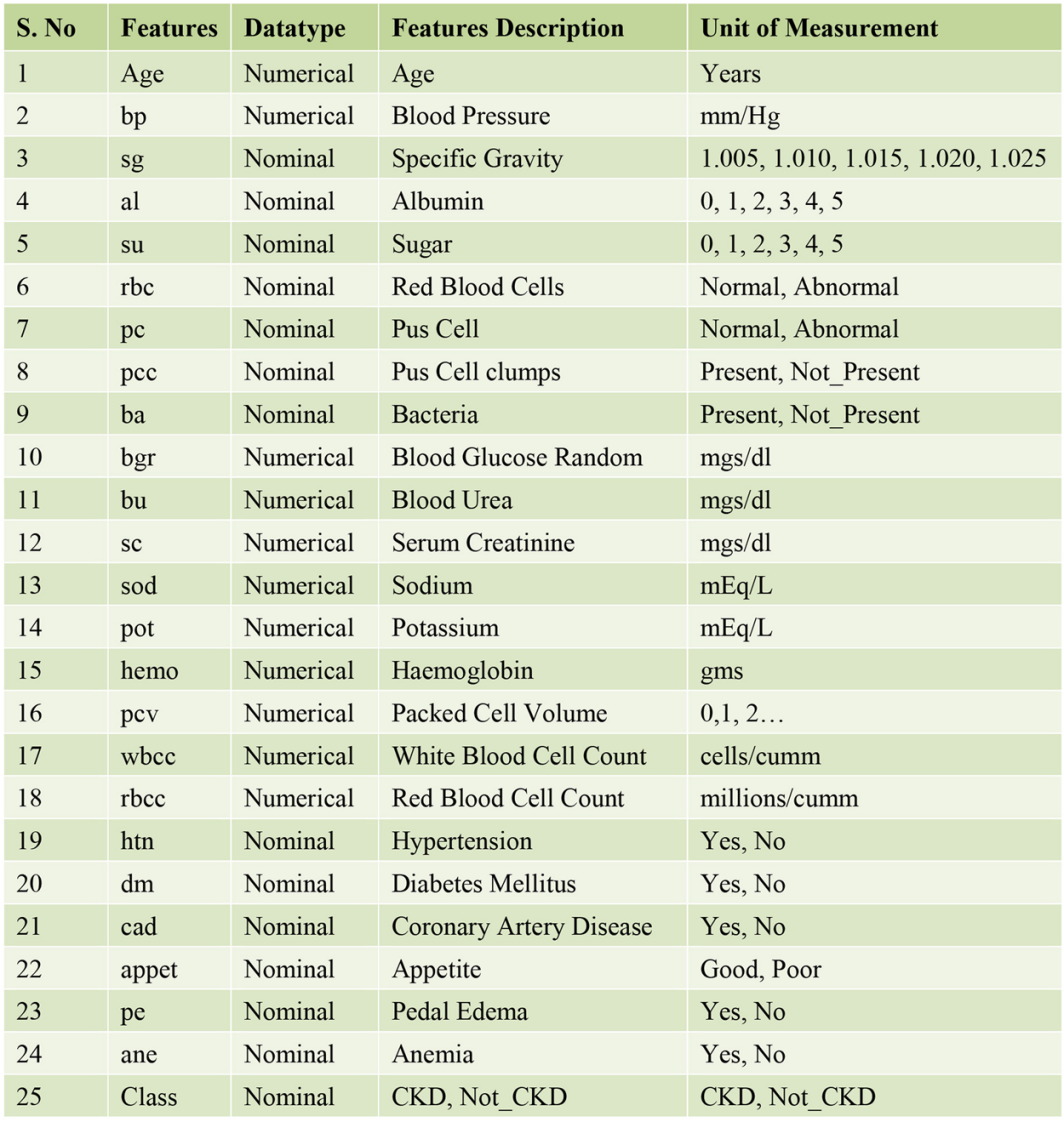
Features that influence on CKD.

For assessing the classifier performance of the D-ACO model, a benchmark CKD dataset^33^ from the UCI repository is used. The dataset description and available features are given in Table 2. The CKD dataset holds a sum of 400 instances with 24 features. Out of the total 400 instances, 250 instances are labeled with CKD present and the rest of the 150 instances are labeled with the non-existence of CKD. The sample frequency distribution and class distribution of the 24 features are shown in Figs 4 and 5 respectively. Besides, the features that influence on CKD are shown in Fig. 6. For experimentation, 10-fold cross validation technique is used to assess the effectiveness of the presented model.

**Table 2:** Dataset Description.

Description	Values
No. of Instances	400
No. of Features	24
No. of Class	2
Percentage of Positive Samples	62.50%
Percentage of Negative Samples	37.50%
Data source	UCI

### Metrics

To highlight the efficiency of the D-ACO algorithm on the CKD dataset, a set of performance metrics include false positive rate (FPR), false negative rate (FNR), sensitivity, specificity, accuracy, F-score and kappa value. In prior to explaining the evaluation parameters, the idea of a confusion matrix is discussed.

**Figure 7:**
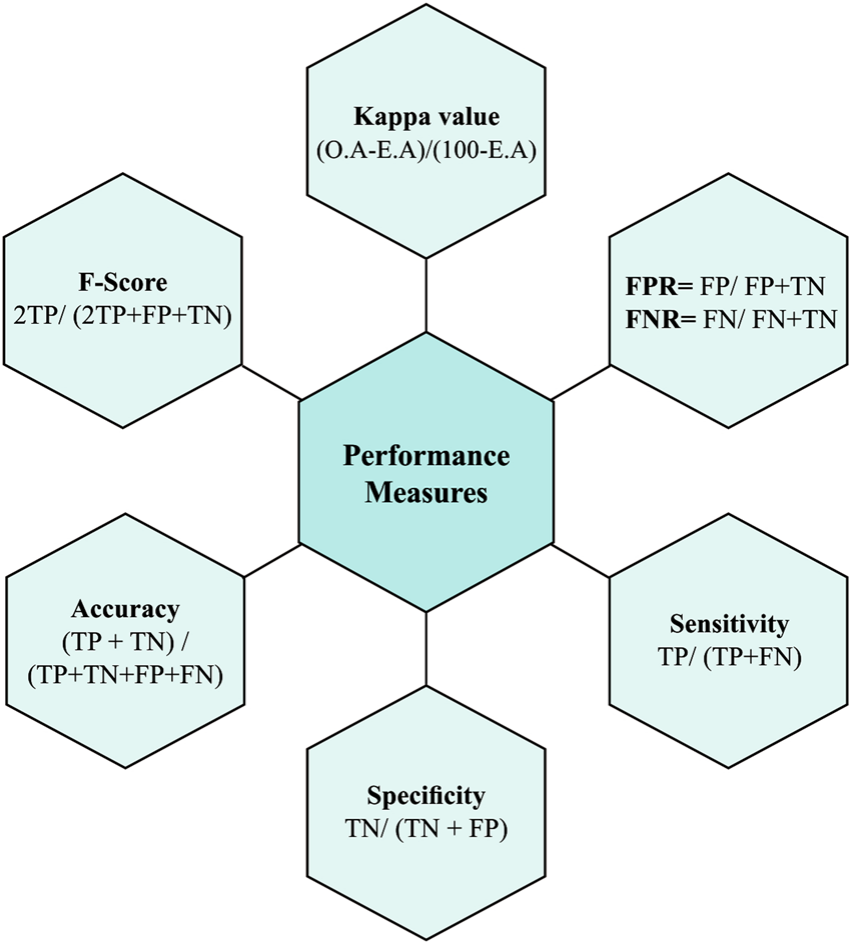
Performance measures. Where, Observed Agreement (O.A) = % (Overall Accuracy). Expected Agreement (E.A) = (% (TP + FP) *% (TP + FN)) + (% (FN + TN)* %(FP + TN)).

Confusion matrix is essential in the assessment of classification performance of any classifiers. It is a 2 × 2 matrix that provides the data about the actual and predicted classifications. The confusion matrix contains four elements: TP, TN, FP and FN. Using these four elements, the classification measures can be defined as given in Fig. 7.

### Results analysis on FS performance

**Figure 8:**
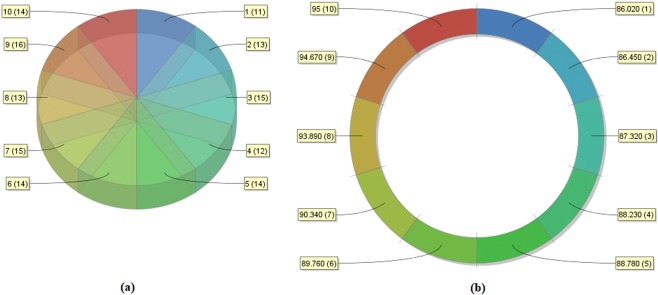
(**a**) Number of chosen features under ten iterations and (**b**) comparing Accuracy of chosen features over ten iterations.

Table 3 provides the obtained FS results obtained by the D-ACO algorithm. Figure 8 shows the number of selected features by D-ACO algorithm over several iterations. The DFS method chooses an average of 14 features from the set of 24 features with a minimum and maximum of 11 and 16 features respectively. At the 10th iteration, the number of selected features is 14. In the same way, from the table, it is observed that the classifier accuracy gradually increasing from 86.02 to 95.00 over the 10 iterations. Using the features selected at the 10th iteration, a maximum classification accuracy of 95 is attained at the 10^th^ iteration.

**Table 3:** Selected Features of CKD using D-ACO.

Features	Iteration 1	Iteration 2	Iteration 3	Iteration 4	Iteration 5	Iteration 6	Iteration 7	Iteration 8	Iteration 9	Iteration 10
Age	✓	—	✓	✓	—	✓	✓	—	✓	—
Blood Pressure	—	✓	—	—	✓	—	—	—	✓	✓
Specific Gravity	✓	✓	✓	✓	✓	✓	✓	✓	—	✓
Albumin	—	—	—	—	—	—	✓	✓	✓	✓
Sugar	—	—	✓	—	—	—	✓	✓	✓	✓
Red Blood Cells	✓	✓	—	✓	✓	✓	✓	—	—	✓
Pus Cell	✓	✓	—	✓	✓	✓	—	✓	✓	—
Pus Cell clumps	✓	—	✓	—	—	—	✓	✓	✓	—
Bacteria	—	✓	✓	✓	✓	✓	✓	✓	✓	✓
Blood Glucose Random	—	✓	✓	✓	✓	✓	✓	✓	✓	✓
Blood Urea	✓	—	—	—	—	—	—	—	—	—
Serum Creatinine	—	✓	—	✓	✓	✓	✓	✓	✓	✓
Sodium	—	—	✓	—	—	—	✓	✓	✓	—
Potassium	—	—	✓	—	—	—	—	—	—	✓
Haemoglobin	✓	✓	—	✓	—	✓	—	✓	—	—
Packed Cell Volume	—	—	✓	—	—	—	✓	✓	✓	—
White Blood Cell Count	✓	✓	✓	✓	✓	✓	✓	—	✓	✓
Red Blood Cell Count	—	—	✓	✓	✓	✓	—	✓	✓	✓
Hypertension	—	✓	—	✓	✓	✓	✓	—	✓	—
Diabetes Mellitus	✓	—	✓	—	—	—	—	✓	—	✓
Coronary Artery Disease	—	✓	✓	—	—	—	✓	—	✓	✓
Appetite	✓	—	✓	✓	✓	✓	✓	—	—	—
Pedal Edema	✓	✓	✓	—	✓	✓	—	—	✓	—
Anaemia	—	✓	—	—	✓	✓	—	—	—	✓

### Comparison of classification performance

Table 4 depicts the rules created by the proposed algorithm and Table 5 shows the comparison of various classification models based on different evaluation parameters.

**Table 4:** Rules produced by the D-ACO Algorithm on CKD dataset.

Rule	Antecedent	Consequent
1	IF rbc = normal AND al < = 0.5	Not_CKD
2	IF bp < = 75.0 AND dm = no	Not_CKD
3	IF wbcc < = 10850.0 AND rbc = normal	Not_CKD
4	IF dm = no AND ane = no	Not_CKD
5	IF ane = no AND pot > 4.15	Not_CKD
6	IF sg < = 1.0175	CKD
7	IF dm = no AND wbcc < = 10850.0 AND rbc = normal	Not_CKD
8	IF bp < = 85.0	Not_CKD
9	IF ba = notpresent AND sg < = 1.0225	CKD
10	IF ane = no AND rbc = normal AND dm = no	Not_CKD
11	IF ba = notpresent AND ane = no	Not_CKD
13	IF sg < = 1.0225 AND ba = notpresent	CKD
14	IF sc > 1.25	CKD

**Table 5:** Performance Evaluation of CKD using D-ACO algorithm with various classifiers.

Performance Measures	Classifiers			
	D-ACO	ACO	PSO	OlexGA
FPR	06.66	15.38	20.00	33.33
FNR	04.00	11.00	12.00	20.00
Sensitivity	96.00	88.88	88.00	80.00
Specificity	93.33	84.61	80.00	66.66
Accuracy	95.00	87.50	85.00	75.00
F-score	96.00	90.56	88.00	80.00
Kappa	89.33	72.06	68.00	46.66

**Figure 9:**
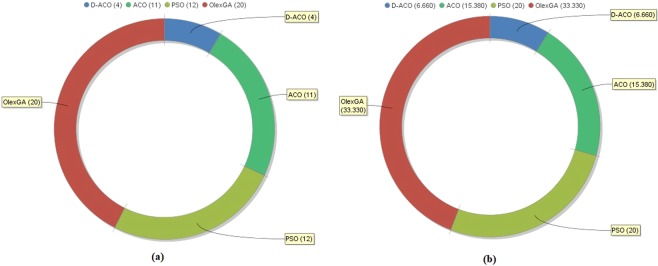
Comparative results of different classifiers in terms of (**a**) FNR and (**b**) FPR.

Figure 9 depicts the obtained results of different classifiers concerning FPR and FNR. From this figure, it is evident that the Olex-GA has minimum FPR and FNR values of 33.33 and 20.0 correspondingly. These values imply that the Olex-GA classification model fails to depict better results on the tested CKD dataset. Likewise, PSO algorithm also attains lower FPR and FNR values of 20.0 respectively. Next, ACO algorithm attains an FPR and FNR value of 15.38 and 11.0. Though ACO algorithm manages to classify data effectively, it fails to show better performance over D-ACO algorithm. On the whole, the proposed D-ACO algorithm has the minimum FPR and FNR value of 6.66 and 4.00 correspondingly.

**Figure 10:**
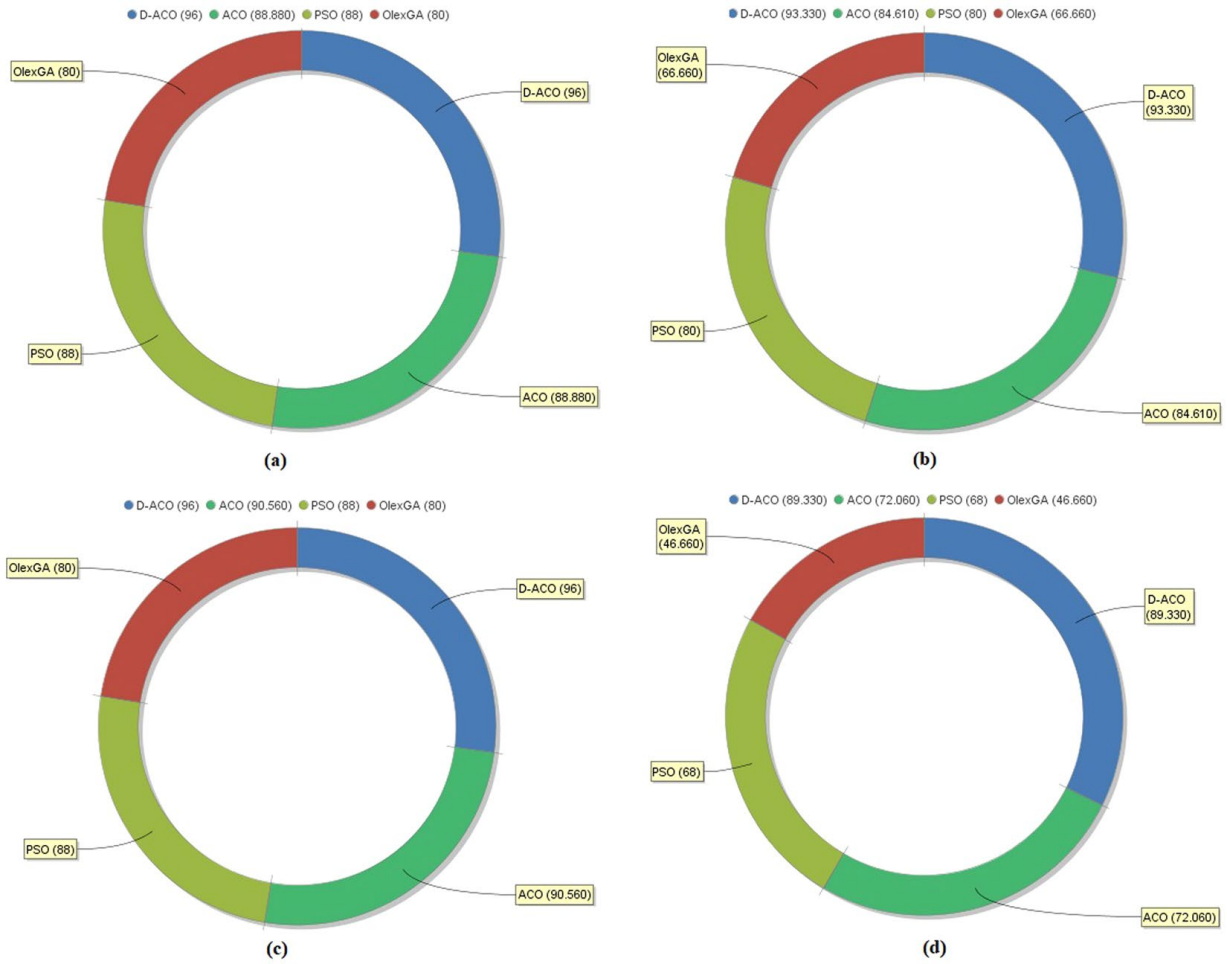
Comparison of various classifiers interms of (**a**) Sensitivity (**b**) Specificity (**c**) F-Score (**d**) Kappa.

The improved results of D-ACO algorithm are due to the addition of DFS which removes the undesirable features to enhance the classifier results. Comparative results of diverse classification models concerning various metrics under different measures are given in Fig. 10. From this figure, it is evident that the Olex-GA attained the sensitivity value of 80.00 which is lesser than the compared ones. Additionally, the ACO and PSO algorithms showed appropriately identical results with sensitivity values of 88.88 and 88.00 correspondingly. The D-ACO model exhibited maximum results with a higher sensitivity value of 96. In the same way, the presented model is found to be effective with a sensitivity value of 96.33 which is higher than the values attained by the compared ones. Likewise, the D-ACO algorithm obtains a maximum F-score of 96 and the order of effective classifiers based on F-score are Olex-GA, PSO and ACO algorithms. Remarkably, the presented D-ACO model shows supreme results over the existing ones in diverse aspects.

**Figure 11:**
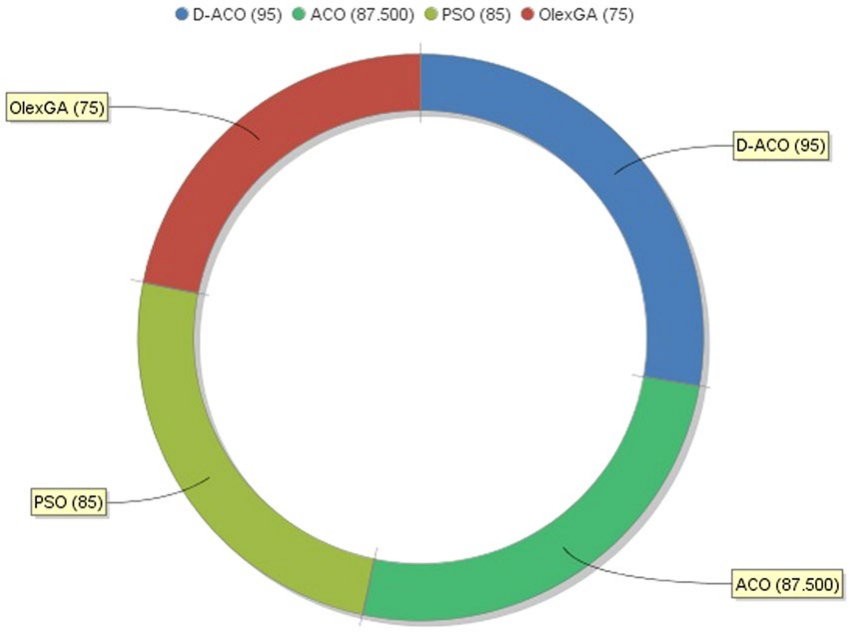
Comparison of various classifiers interms of accuracy.

The most important measure of the classification algorithm is accuracy, and the comparative results based on accuracy is given in Fig. 11. Among the three compared classification algorithms with D-ACO algorithm, the Olex-GA achieves the lowest accuracy of 75 implying the poor classifier results. However, the ACO and PSO algorithms perform well and show competitive performance over one another. Though ACO and PSO algorithms have attained an accuracy of 87.5 and 85, they failed to shows superior performance to D-ACO algorithm. The maximum accuracy of 95 is obtained by the D-ACO algorithm indicating the effective performance on the employed CKD dataset.

Finally, the results of different classification algorithms on CKD dataset interms of various performance measures revealed that the proposed D-ACO algorithm is found to be efficient on the classification of CKD dataset. This is due to the advantage of DFS as well as the nature of wrapper method which continuously execute the DFS and ACO algorithms consecutively.

## Conclusion

This paper has presented an intelligent prediction and classification system for healthcare, namely DFS with ACO algorithm called D-ACO algorithm is proposed for the classification of CKD dataset. The proposed D-ACO framework, however, jointly performs FS, ACO based learning and removes irrelevant features. Using a benchmark CKD dataset, the efficiency of the D-ACO algorithm is evaluated, and a comparison is also made with the existing methods. On comparing with the existing methods, the proposed D-ACO algorithm outperformed the other methods with improved classification performance in various aspects. In overall, the proposed D-ACO algorithm is found to be an appropriate classifier for the identification of the CKD.

## Data Availability

The dataset generated analyzed during the current study are available in the UCI repository, [Chronic kidney disease dataset, available at https://archive.ics.uci.edu/ml/datasets/chronic_kidney_disease].
