# Author Correction: Intelligent Diagnostic Prediction and Classification System for Chronic Kidney Disease

**DOI:** 10.1038/s41598-020-61542-w

**Published:** 2020-03-06

**Authors:** Mohamed Elhoseny, K. Shankar, J. Uthayakumar

**Affiliations:** 1 Faculty of Computers and Information, Mansoura University, Mansoura, Egypt; 2 School of Computing, Kalasalingam Academy of Research and Education, Krishnankoil, India; 3 Department of Computer Science, Pondicherry University, Pondicherry, India

Correction to: *Scientific Reports* 10.1038/s41598-019-46074-2, published online 03 July 2019

In Algorithm I, some steps are missing after line 16. The correct Algorithm I appears below.

**Algorithm I:**
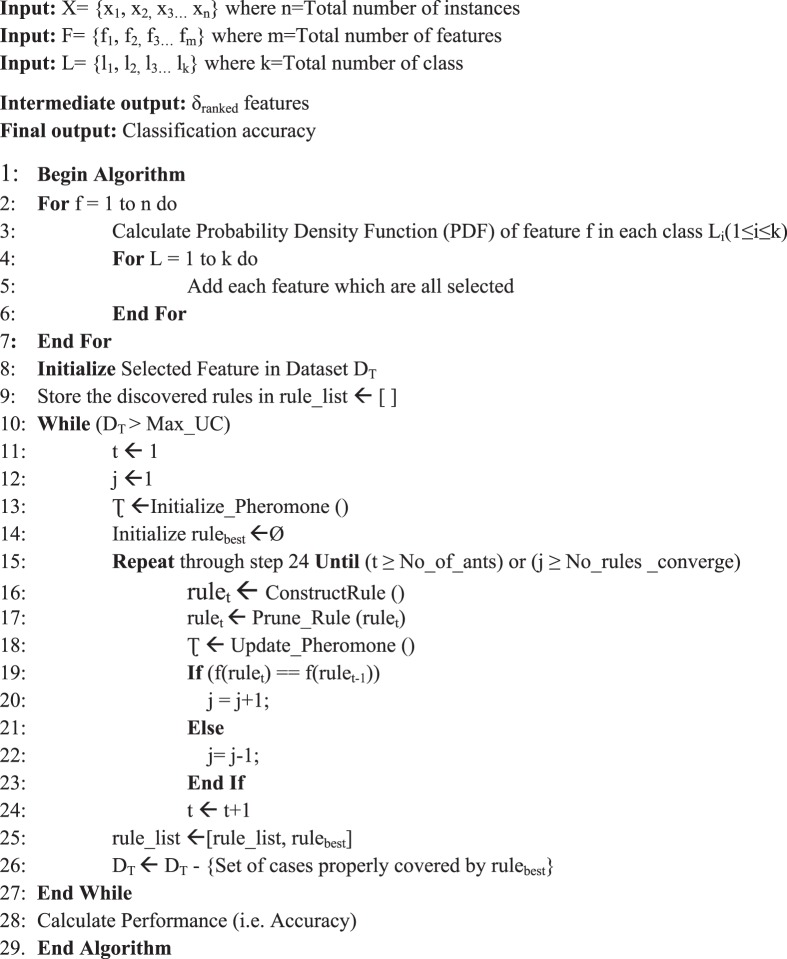
Density based feature selection with Ant Colony Optimization (D-ACO) for Data Classification.

